# Endoscopic procedures for removal of foreign bodies of the aerodigestive tract: The Bugando Medical Centre experience

**DOI:** 10.1186/1472-6815-11-2

**Published:** 2011-01-21

**Authors:** Japhet M Gilyoma, Phillipo L Chalya

**Affiliations:** 1 Department of Surgery, Weill- Bugando University College of Health Sciences, Mwanza, Tanzania

## Abstract

**Background:**

Foreign bodies in the aerodigestive tract continue to be a common problem that contributes significantly to high morbidity and mortality worldwide. This study was conducted to describe our own experience with endoscopic procedures for removal of foreign bodies in the aerodigestive tract, in our local setting and compare with what is described in literature.

**Methods:**

This was a prospective descriptive study which was conducted at Bugando Medical Centre between January 2008 and December 2009. Data were collected using a structured questionnaire and analyzed using SPSS computer software version 15.

**Results:**

A total of 98 patients were studied. Males outnumbered females by a ratio of 1.1:1. Patients aged 2 years and below were the majority (75.9%). The commonest type of foreign bodies in airways was groundnuts (72.7%) and in esophagus was coins (72.7%). The trachea (52.2%) was the most common site of foreign body's lodgment in the airways, whereas cricopharyngeal sphincter (68.5%) was the commonest site in the esophagus. Rigid endoscopy with forceps removal under general anesthesia was the main treatment modality performed in 87.8% of patients. The foreign bodies were successfully removed without complications in 90.8% of cases. Complication rate was 7.1% and bronchopneumonia was the most common complication accounting for 42.8% of cases. The mean duration of hospital stay was 3.4 days and mortality rate was 4.1%.

**Conclusion:**

Aerodigestive tract foreign bodies continue to be a significant cause of childhood morbidity and mortality in our setting. Rigid endoscopic procedures under general anesthesia are the main treatment modalities performed. Prevention is highly recommended whereby parents should be educated to keep a close eye on their children and keep objects which can be foreign bodies away from children's reach.

## Background

Foreign bodies in the aerodigestive tract are an important cause of morbidity and mortality in the two extremes of life and pose diagnostic and therapeutic challenges to otorhinolaryngologists [1]. The ingestion and inhalation of foreign bodies occurs most commonly in children's population, especially in their first six years of life [2,3], with a peak incidence in children between 1 and 3 years [1,4]. Children are naturally susceptible to be involved in FB injuries due to lack of molar teeth, the tendency to oral exploration and to play during the time of ingestion, and the poor coordination of swallowing [4,5]. On the other hand, the elderly are those with thoracic neurological disease, decreased gag reflex due to alcohol seizures, stroke, Parkinsonism, trauma and senile dementia [6].

The accurate diagnosis of aerodigestive tract foreign bodies may be missed even by an experienced clinician. The delayed symptoms of foreign body in the aerodigestive tract may mimic other common conditions like asthma, recurrent pneumonia, upper respiratory infection and persistent cough [1,7-10].

Foreign bodies in the aerodigestive tract present with a wide spectrum of clinical presentation, patients often present in the emergency with acute onset respiratory distress and occasionally in a cyanosed state. At the other end of the spectrum is the patient presenting with nothing more than a history of aspiration and on investigation is found to have a foreign body in his aerodigestive tract [10].

The symptoms and signs produced depend upon the nature, size, location and time since lodgment of the foreign body in the aerodigestive tract. A large foreign body occluding the upper airway or esophagus may lead to severe symptoms and even sudden death whereas a small foreign body lodged in the aerodigestive tract may present with less severe symptoms [10,11].

Foreign bodies lodged in the esophagus for a long time may be associated with complications such as mucosal ulceration, esophageal obstruction, perforation, intrinsic stenosis and esophageal diverticulum [12], whereas foreign bodies retention in the airway may lead to complications such as severe respiratory distress, lung collapse and recurrent chest infection [13]. Early diagnosis and treatment are imperative to prevent mortality as well as complications.

There is paucity of local data regarding the management of foreign bodies in the aerodigestive tract as there is no study which has been done in our setting or any other hospital in the country. This study was done in our setting to describe our experience with endoscopic procedures for the removal of foreign bodies in the aerodigestive tract, with a review of the pertinent literature.

## Methods

This was a prospective descriptive study which was conducted at the Accident and. Emergency department of Bugando Medical Centre (BMC) over a 2-year period between January 2008 and December 2009. BMC is a 1000-bed, tertiary care and teaching hospital for the Weill-Bugando University College Health Sciences (WBUCHS). The study subjects included all patients of all age groups and gender who presented to the A &E department with history of foreign bodies in the aerodigestive tract. Patients with history of foreign bodies in the aerodigestive tract but could not be identified at endoscopic examination and those who died before endoscopic procedures were excluded from the study. All patients included in the study were, after informed written consent to participate in the study, enrolled in the study. Approval to conduct the study was sought from the WBUCHS/BMC joint institutional ethic review committee before the commencement of the study.

All patients with history of foreign bodies in the aerodigestive tract were subjected to endoscopic examinations (oesophagoscopy or bronchoscopy). Data were collected using a structured, pre-tested and coded questionnaire. Included in questionnaire were; age, sex, area of residence, family history of foreign bodies in the aerodigestive tract, the type of foreign body, anatomical location of the foreign body, treatment and outcomes (length of hospital stay, mortality & postoperative complication). Data were analyzed using SPSS computer software version 15.

## Results

During the period under study, a total of 98 patients with established foreign body in the aerodigestive tract were studied. 52 (53.1%) were males and females were 46 (46.9%) with a male to female ratio of 1.1:1. Their ages ranged from 1 year to 63 years (mean 7.04 ± 14.62 years). The median was 2 years. Patients aged ten years and below were the majority and accounted for 87 (88.8%). Of these, 66 (75.9%) patients were aged two years and below. The majority of patients 64(65.3%) were from the urban areas around Mwanza city. No patient had family history of foreign body in the aerodigestive tract (Table 1).

**Table 1:** Patients characteristics

Patients characteristics	Number of patients	Percentage
**Age (years)**		
° ≤ 10	87	88.8
° > 10	11	11.2
**Sex**		
° Males	52	53.1
° Females	46	46.9
**Area of residence**		
° Urban	64	65.3
° Rural	34	34.7
**Family history of foreign bodies**		
° Yes	-	-
° No	98	100

Patients with foreign bodies in the esophagus were the majority accounting for 54 (55.1%) of cases, whereas patients with foreign bodies were 44(44.9%). Sixty-three (64.3%) patients presented to hospital within 24 hours, whereas 20 (20.4%) presented between 1 day to 7 days and the remaining 15(15.3%) presented to hospital after 7 days. The most common reasons for delay presentation were lack of money for transport and inappropriate diagnosis and treatment given in the peripheral hospitals. A positive history of foreign body in the aerodigestive tract were recorded in 92 (93.9%) of cases, whereas in the remaining 6 (6.1%) patients the diagnosis of foreign bodies in the aerodigestive tract was made based on clinical presentation and radiological investigation on admission. Sixty-eighty (69.4%) of the patents were asymptomatic on admission despite positive history of foreign bodies in the aerodigestive tract. The most common clinical presentations were cough, wheezing, dyspnea, choking, vomiting, and drooling of saliva and difficulty in swallowing. Coins were the most common type of foreign body in the esophagus accounting for 72.2% of patients, whereas groundnuts were the most common type of foreign body in the airway accounting for 72.7%. The type of foreign bodies in the aerodigestive tract (airway & esophagus) is shown in Table 2 &3 respectively.

**Table 2:** The type of foreign bodies in the airways

The type of foreign body	Number	Percentages
Groundnuts	32	72.7
Plastic objects	6	13.6
Bead	4	9.1
Fish bone	1	2.3
Stone	1	2.3
**Total**	44	100

**Table 3:** The type of foreign bodies in the esophagus

The type of foreign bodies	Number	Percentages
Coins	39	72.2
Fish bone	6	11.1
Coca-cola cover	3	5.6
Battery cover	2	3.7
Pin	1	1.9
Chicken bone	1	1.9
Meat bolus	1	1.9
Metal wire	1	1.9
**Total**	54	100

The trachea was the most common site of foreign body's lodgment in the airways accounting for 52.2% of cases, whereas cricopharyngeal sphincter was the commonest site in the esophagus in 68.5% of cases. Table 4 shows the anatomical location where foreign bodies were lodged in the aerodigestive tract.

**Table 4:** Anatomical location where foreign bodies were lodged in the aerodigestive tract (N = 98)

Anatomical location	Number	Percentages
**In the airway**	**44**	**44.9**
° Glottis	9	20.5
° Trachea	23	52.2
° Bronchus	12	27.3
**In the esophagus**	**54**	**55.1**
° Cricopharyngeal sphincter	37	68.5
° Bifurcation of trachea	17	31.5

In the bronchus, the right bronchus was the most common site of foreign body impaction in 9 (75%) of cases and the remaining 3(25%) were in the left bronchus. Plain neck/chest x-rays reviewed radiopaque foreign bodies in 55 (56.1%) of cases. Rigid endoscopy (oesophagoscopy and bronchoscopy) with forceps removal under general anesthesia was the main treatment modality performed in 86(87.8%) of patients. In 9(9.2%) patients, the foreign bodies especially in the upper esophagus were removed by Magill forceps extraction. Foley's catheter without fluoroscopic control was used to remove esophageal foreign bodies in the remaining 3(3.1%) patients. The foreign bodies were successfully removed without complications in 89 (90.8%) of cases. A total of 67 (68.4%) patients especially those who had bronchoscopy required at least an overnight hospitalization to be able to monitor immediate postoperative complications resulting from the procedure and anesthesia. The remaining 31(31.6%) were treated as outpatients. Seven post operative complications were recorded giving a complication rate of 7.1%. Postoperative complications are shown in Table 5.

**Table 5:** Postoperative Complications

Complications	Number	Percentages
Bronchopneumonia	3	42.8
Bronchopneumonia	2	28.6
Esophageal stricture	1	14.3
Severe respiratory distress	1	14.3
**Total**	**7**	100

The majority of in-patients were discharged between 1 day and 7 after foreign body removal. The overall duration of hospital stay of in-patients ranged from 1 day to 13 days (mean 3.4 days). Four patients died giving a mortality rate of 4.1%. The most common causes of death were cardiopulmonary arrest, severe respiratory distress and severe pneumonia (*p *< 0.001).

## Discussion

Foreign bodies lodged in the aerodigestive tract are a common surgical emergency presenting to the Accident & Emergency department in many centres and contribute significantly to high morbidity and occasionally mortality [1]. Children aged between 1 and 3 years are commonly affected [1,4]. In the present study, the majority of patients were children aged two years and below which is in agreement with other studies [1,4,14,15]. Several factors contribute to high incidence of aerodigestive tract foreign bodies in this age group including social factors (e.g. carelessness of parents, children's habit of putting objects in their mouth, crying/playing during eating) and anatomical factors (e.g. absent of molar teeth, inadequate control of deglutition) have been mentioned [16-18].

In our study, males were slightly more affected than females with a male to female ratio of 1.1:1 which is in agreement with other studies [15,19,20]. The reasons for the male preponderance in our study may be attributed to the overactive nature of male babies as compared to the females.

In the present study, a positive history of foreign body in the aerodigestive tract was recorded in 93.9% of cases and 69.4% of these were found to be asymptomatic on admission which is comparable to other studies [1,8,9,16]. Cohen [21] has strongly advocated that all patients presenting with positive history of foreign body in the aerodigestive tract, even when the physical finding and radiological examinations is negative must be subjected to endoscopic evaluation. In the present study, all patients with a positive history of foreign body in the aerodigestive tract were subjected to endoscopic removal.

The commonest foreign bodies found in our study were coins and groundnuts in the esophagus and airways respectively, which is similar to findings reported by other studies [22,23]. The reason for high incidence of these foreign bodies in our study is due to the fact that these commodities are widely used in this area. The preponderance of the coins may also be attributed to the free access children have to coins in our environment, which are usually given as gifts.

The trachea was the most common site of foreign body's lodgment in the airways and cricopharyngeal sphincter was the commonest site in the esophagus. Similar foreign body's lodgment pattern was also reported by others [1,22,23]. In the bronchus, the majority of foreign bodies in our study come to rest in the right bronchus which is agreement with other authors [1,16,24]. This observation is attributed to the fact that the right bronchus is more vertical and wider than the left ones.

The majority of our patients presented to the A & E department within 24 hours of inhalation/ingestion of foreign which is similar to other reports [22,23]. Our experience shows that early presentation is common with very young children, and when there are more serious symptoms of respiratory distress and swallowing difficulty, thus compelling the frightened patients or parents to seek medical attention. Late presentation is more common in asymptomatic cases.

Radiography plays a vital role in the diagnosis of radio-opaque foreign body in the aerodigestive tract. In agreement with other series [2,22,23], the plain radiography of chest/neck in our study detected foreign bodies in the aerodigestive tract in 56.1% of cases. This percentage is high enough to warrant radiographic surveillance of all patients presenting with history of foreign body in the aerodigestive tract. However, a negative radiographic result does not exclude the presence of foreign bodies in the aerodigestive tract as radio-lucent objects like rubber materials, groundnuts and bolus of meat are not easily detected by plain radiography.

Endoscopic removal of foreign bodies in the aerodigestive tract using rigid scopes under general anesthesia has been reported to be a golden standard procedure [22-28]. Rigid endoscopy, as compared to flexible endoscopy is a useful method to diagnose and remove foreign bodies in the aerodigestive tract as it has a large lumen and allows better visualization of the potential anatomic sites of foreign body impaction in the aerodigestive tract [29]. However, the procedure is not without risks especially perforation which has a high morbidity and potential mortality. Besides the surgical risks the patients is also subject to anesthetic risks. Other treatment modalities in the removal of foreign bodies in the aerodigestive tract include use of Magill forceps and Foley's catheter in the removal of foreign bodies in the esophagus [30,31]. In the present study, rigid endoscopy (oesophagoscopy and bronchoscopy) with forceps removal under general anesthesia was the main treatment modality performed which conforms with others studies [22-28]. In the view of potential complications resulting from rigid endoscopic procedures and the use of general anesthesia, our patients required at least an overnight hospitalization so as to monitor these complications. Magill forceps extraction and Foley's catheter without fluoroscopic control were used to remove esophageal foreign bodies in 9.2% and 3.1% of cases respectively. It is therefore recommended that in places where rigid endoscopy is not available like in most peripheral hospitals, Magill forceps and Foley's catheter without fluoroscopic control can safely be used in the removal of foreign bodies in the esophagus.

In our study, the foreign bodies were successfully removed without complications in 90.8% of cases which is similar to other studies reported elsewhere [22-28]. However, the complication and mortality rates in our study were found to be higher than that reported in other studies [28-31]. The reasons for this observation could be as a result of either of the two reasons. First, the removal of foreign bodies in the aerodigestive tract were often performed or attempted by the inexperienced resident doctors who were the first on call. This observation calls for urgent training of our resident doctors on how to perform these procedures and that only experienced endoscopist should be allowed to perform endoscopic procedures for the removal of foreign bodies in the aerodigestive tract. Secondarily, some patients presented for the foreign body removal only after a failed, traumatic attempts in peripheral hospitals in hands of inexperienced operators. However, it should be kept in mind that rigid endoscopic procedures (oesophagoscopy and bronchoscopy) are difficult procedures even in experienced hands.

## Conclusion

Foreign bodies in the aerodigestive tract are among the most common causes of surgical emergencies presenting to the Accident & Emergency department of BMC and contribute significantly to high morbidity and occasionally mortality. Children aged two years and below are commonly affected. Rigid endoscopies with forceps removal under general anesthesia are the preferred management modality. It is recommended that the removal of foreign bodies in the aerodigestive tract should only be performed or attempted by experienced endoscopists. Since aerodigestive tract foreign bodies are preventable surgical condition, preventive measures should be directed at the high risk group (children) whereby parents should be educated to keep a close eye on their children and keep objects which can be foreign bodies away from children's reach.

## Competing interests

The authors declare that they have no competing interests.

## Authors' contributions

JMG conceived the study and did the literature search, coordinated the write-up, editing. PLC participated in the literature search, writing of the manuscript, editing and submission of the article. All the authors read and approved the final manuscript

## Authors' information

JMG: Senior Consultant General/ENT surgeon, Senior Lecturer and Head, Department of Surgery, Well Bugando University Collage of Health Sciences.

PLC: Consultant general surgeon and Lecturer, Department of Surgery, Well Bugando University Collage of Health Sciences.

## Pre-publication history

The pre-publication history for this paper can be accessed here:

http://www.biomedcentral.com/1472-6815/11/2/prepub
